# Supplementary material for: Are carbon emissions trading and green financial instruments synergistic? -Comprehensive quantitative research based on content analysis
Source: PLoS One. 2024 Mar 7;19(3):e0298601. doi: 10.1371/journal.pone.0298601 (PMC10919601; doi:10.1371/journal.pone.0298601)
Supplement: S1 Data — (ZIP) [file pone.0298601.s001.zip › Data/地级市Z_F工作报告.zip!/╜¡╦╒/│ú╓▌╩╨/└┤╫╘╣1⁄2╓┌║┼í╛┬φ┐╦╩2╛▌═°í┐_2018.docx]

各位代表：

　　现在，我代表常州市人民政府向大会报告政府工作，请予审议，并请市政协各位委员和其他列席人员提出意见。

　　一、2017年工作回顾

　　过去一年，面对严峻复杂的宏观形势和艰巨繁重的改革发展任务，市政府在中共常州市委的坚强领导和市人大、市政协的监督支持下，紧紧依靠全市人民，深入贯彻习近平新时代中国特色社会主义思想，扎实推进“两聚一高”新实践，全力增创发展新优势、种好常州幸福树，顺利完成市十六届人大一次会议确定的各项任务，实现了本届政府的良好开局。

　　（一）大力振兴实体经济，经济发展量质齐升。主要经济指标增幅在全省位居前列，预计全年完成地区生产总值6580亿元，增长8%左右；完成一般公共预算收入518.8亿元，增长8%；完成固定资产投资3890亿元，增长8%左右，其中工业投入2100亿元，增长9%左右；完成社会消费品零售总额2434亿元，增长10.5%；进出口总额突破300亿美元，增长12%；实际到账外资25.5亿美元；工业应税销售、金融机构本外币存款余额首次超过万亿元。扎实开展“重大项目提升年”活动，重大项目建设取得新突破，省重大项目开工数全省第一，省市重点项目开工率双双实现100%，全年新增重大签约项目23个，其中时代上汽先进动力电池及系统化等5个项目超100亿元或10亿美元；同方威视制造基地、常州检验检测认证产业园一期等41个省市重点项目竣工投用。坚持把企业上市作为增创发展新优势的关键一招，出台“十六条”新政和三年行动计划，企业上市创历史最好成绩，新增上市公司12家，总数达55家；新增新三板挂牌企业30家，总数达124家。高度重视发展民营经济，实施企业培育、产业升级等九大行动，民间投资增长12%左右，规模以上民营工业企业利润总额增长15%左右；全市新设市场主体91670户，注册资本（金）2205.2亿元，分别增长28.7%、15%，实有市场主体总数突破50万户。

　　（二）深入实施转型创新，新旧动能加快转换。扎实推进苏南国家自主创新示范区和“中国制造2025”苏南城市群试点示范建设，出台科技创新“1+3”新政。成功承办世界石墨烯创新大会。常州科教城连续4年位列中国最佳创业园区第二名，获评省优秀科技园区和省知识产权服务集聚区。全市新增省级以上企业研发机构50家、省级以上众创空间23家，新增31家国家两化融合管理体系贯标评定企业、数量全省第一。国家工业强基工程项目累计达10个、列全国地级市第二。预计全社会研发经费支出占地区生产总值比重达2.79%左右。改革创新“龙城英才计划” ，新签约引进领军型创新创业人才超200名，新入选国家“千人计划”3人、“万人计划”1人，入选省“双创”团队7个，每万名劳动者中高技能人才数连续3年位列全省第一，我市选手宋彪成为我国获得世界技能大赛最高奖阿尔伯特大奖第一人。深入实施“三位一体”工业转型升级战略，十大产业链产值增长15%左右，占规模以上工业比重达36%以上，新能源汽车及汽车核心零部件、新材料、智能制造装备产业链产值均增长20%左右；高新技术产业产值占规模以上工业比重达45%左右。加快服务业创新发展，服务业增加值增长10%左右，占地区生产总值比重达51%左右；数码大方、航天云网、佰腾科技专利云平台等“互联网+”新业态不断涌现；旅游总收入、接待国内外游客预计分别达937亿元、6536万人次，春秋淹城荣膺国家5A级景区；常州征信平台加快建设，常州创业创新服务平台建成运行。扎实推进农业现代化，国家现代农业示范区建设水平位居全国地级市首位，启动创建国家农产品质量安全市，高标准农田、高效设施农业面积比重分别超过62%、22%，新增2个全国“一村一品”示范村镇。粮安保障得到加强，供销社改革取得新进展。

　　（三）全面深化改革开放，发展活力明显增强。深入推进供给侧结构性改革，“地条钢”整治工作通过国家验收，压减水泥产能120万吨，降低企业成本160亿元左右。不断深化产城融合综合改革，19个市级试点区域建设取得新进展。市级投融资平台完成整合，向实体化市场化集团化方向转型发展。完成市、区财政管理体制调整，市、区统筹发展能力进一步提升。深入推进农村改革，完成农村土地承包经营权确权登记颁证工作，武进区农村土地制度“三项改革”试点在全国实现“七个率先”，雷利电机成为全国首家以农村集体建设用地入市的上市公司。不断提升对外开放水平，新增世界500强投资项目6个、跨国公司地区总部和功能性机构4家；获批开通进境粮食、冰鲜水产品指定口岸，跨境电商公共服务平台等建成投用；常州机场新开通4条境外航线、全年旅客吞吐量达251万人次。大力推进功能载体建设，常州高新区、武进高新区在全国国家高新区综合排名分别上升8位、3位，常州经开区建设步伐加快，天宁开发区东扩、钟楼开发区西进全面实施，苏澳合作园区筹建工作取得阶段性成果；2家单位被列为省级军民结合产业示范基地；孟河镇入选全国特色小镇，石墨烯小镇、智能传感小镇、殷村职教小镇入选省首批特色小镇。

　　（四）加快建设美丽常州，城乡面貌持续优化。启动新一轮城市总体规划修编。加快重大交通设施建设，溧高高速、常宜高速开工建设，233国道金坛段、360省道溧阳段等工程有序推进，长虹西路快速化改造基本完成。不断完善城市路网，建成飞龙西路西延、惠国路、芦墅路等城市道路，地铁1、2号线工程加快实施。常州污泥焚烧中心一期开工建设。新沟河、新孟河延伸拓浚工程进展顺利。电力通信等基础设施加快建设。扎实推进城乡一体化，美丽乡村示范项目建设全面展开，7个村镇被评为全国文明村镇，竹箦镇陶庄村入选全国改善农村人居环境示范村，5个村入围首批省特色田园乡村试点，溧阳1号旅游公路成为省首批旅游风景道。

　　加大城市环境综合整治力度，道路交通秩序综合治理取得阶段性成效，国家餐厨废弃物资源化利用试点城市通过验收。扎实开展 “263”专项行动，累计关停化工企业315家、关闭搬迁畜禽养殖场2583家，长江水上过驳专项整治扎实有效，魏村水源地达标整治基本完成，市区空气质量优良率达68.2%，市区PM2.5平均浓度下降7.9%，省级以上水环境考核断面消除劣Ⅴ类水体、优Ⅲ类比例达63.6%。大力推进生态绿城和森林城市建设，建成皇粮浜湿地公园、30个社区体育公园等项目，成片造林1.7万亩。完成省政府节能降耗总量、强度“双控”目标，成为国家低碳试点城市。

　　（五）更加聚焦富民惠民，民生福祉不断改善。坚持民生优先，制定实施富民增收“1+3+3”文件。民生支出占一般公共预算支出的比例达80%。居民收入稳定增长，预计全体居民人均可支配收入达41740元，增长8.6%，居民消费价格总水平涨幅控制在2%左右。加大精准扶贫力度，第四轮茅山老区“百千万”帮扶工程取得明显成效，经济薄弱村村级总收入全部超过100万元。积极促进就业创业，全市城镇新增就业13.5万人，扶持创业1.7万人，城镇登记失业率控制在4%以内。稳步提高社会保障水平，全市低保标准提高至760元/月，实现大市范围内同城同标。持续扩大住房保障覆盖面，共有产权房试点稳步推进，各类棚户区改造新开工25757套（户）、基本建成19768套（户），住房保障全覆盖工程获中国人居环境范例奖。深入推进教育教学改革，教育现代化水平保持全省第一方阵，24所新建、改扩建学校竣工，学生、社会对学校满意度等5项调查均列全省第一，学前教育综合改革取得新进展。全面启动“健康常州”建设，综合医改深入推进，建成多种类型医联体120多个，常州市、金坛区获全省家庭医生签约服务十大创新举措奖，国家健康医疗大数据常州中心及产业园加快建设。深入实施幸福康养示范工程，成为国家康复辅助器具产业综合创新试点区，标准化居家养老服务中心（站）城乡覆盖率分别达60%、36%。不断完善公共文化服务体系，常州文化广场项目顺利推进，建成330个基层综合性文化服务中心。全面加强历史文化名城保护，青果巷历史文化街区和张太雷、唐荆川、周有光等名人馆加快建设。《常州市志（1986—2010）》成功入选“中国志书精品工程”。同步推进竞技体育和群众体育，建成天宁区、钟楼区全民健身中心，常州运动员在第十三届全运会上取得历史最好成绩。大力提升文明城市建设水平，溧阳获评全国文明城市，常州成为全国首批全域文明城市。扎实开展平安常州建设，十九大安保成绩突出，政法队伍满意率全省第一，群众安全感位居全省前列，获评全国社会治安综合治理优秀市，在全省设区市中唯一蝉联“长安杯”。安全生产工作扎实有力，防震减灾、应急管理、质监食药监、海关国检、海事边防工作水平不断提升。着力办好民生实事，“三优三安两提升”实事项目全面完成。国防动员、双拥工作进一步加强，民族宗教、外事侨台、档案、妇女儿童、老龄、残疾人和关心下一代等工作取得新成效。社会大局保持和谐稳定。

　　（六）加强政府自身建设，行政效能得到提升。稳步推进相对集中行政许可权和承担行政职能事业单位改革试点，武进区、常州高新区、常州经开区行政审批局挂牌运行。有序推进政务服务“一张网”建设和“不见面审批”改革，在全省率先公布建设项目中介服务事项清单，“3550”改革初见成效。新政务服务中心建成投运，服务效能明显提高。进一步加强作风建设，“三大一实干”活动取得显著成效，“常州政企通”实现24小时在线服务。深化政务公开，开展基层政务公开标准化规范化试点，“阳光扶贫”和“三资三化”专项提升扎实推进。加强法治政府建设，建立市政府常务会议学纪学法制度，全面落实法律顾问制度，综合行政执法体制改革全省领先。强化科学决策，组建发展战略特聘顾问团队。审计统计、司法行政等工作取得新业绩。自觉接受市人大、市政协及各民主党派的监督，办理人大代表建议、政协提案521件，妥善解决了一批经济社会民生问题。严格落实党风廉政建设主体责任，政府系统反腐倡廉工作取得实效。

　　各位代表，过去的一年，发展成就令人鼓舞。这是全市人民在中共常州市委正确领导下凝心聚力、团结奋斗的结果，是市人大、市政协加强监督、鼎力支持的结果。在此，我代表市人民政府，向辛勤工作在各个领域的全市人民，向市人大代表、政协委员，向市各民主党派、工商联、人民团体、各界人士和离退休老同志，向驻常人民解放军、武警官兵、公安政法干警和部省驻常单位，向所有关心、支持、参与常州建设和发展的海内外朋友们，致以崇高的敬意和衷心的感谢!

　　在肯定成绩的同时，我们也清醒地看到，当前我市发展仍面临不少困难和问题：发展不充分、不平衡、不协调的问题依然突出，发展质量和效益有待进一步提高；产业创新能力仍显不足，重点领域改革和开放还要进一步提速；资源环境压力仍然较大，生态环境保护和资源节约集约利用需要持续用力；民生工作与群众的期望还有差距，基本公共服务标准化、均等化、优质化水平有待提升；少数部门和工作人员服务意识不强、担当精神不足，作风建设、反腐倡廉建设仍需进一步加强。我们对此高度重视，将采取切实有效措施认真加以解决，不辜负全市人民的期待。

　　二、2018年工作安排

　　今年是全面贯彻党的十九大精神的开局之年，是改革开放40周年，也是决胜高水平全面建成小康社会、实施“十三五”规划承上启下的关键之年。今年政府工作的总体要求是：全面贯彻党的十九大精神，以习近平新时代中国特色社会主义思想为指导，认真落实中央、省和市委的决策部署，坚持稳中求进工作总基调，坚持新发展理念，按照高质量发展的要求，以供给侧结构性改革为主线，以“重大项目增效年”活动为主题，持续深化增创发展新优势、种好常州幸福树“六大行动”，统筹做好稳增长、促改革、调结构、惠民生、防风险各项工作，努力实现经济社会持续健康发展，推动“强富美高”新常州建设取得更大进展。

　　做好今年和今后一个时期的工作，必须做到“五个坚持”：

　　一是坚持把人民群众对美好生活的向往作为奋斗目标。牢固树立以人民为中心的发展思想，谋划好富民惠民的新思路、新举措。坚持尽力而为、量力而行，着力解决好发展不平衡不充分问题，在扩大优质教育覆盖面、夯实家庭医生签约服务、满足群众多层次养老需求、改善市民居住环境和出行条件、提高大病医疗救助水平等方面下更大力气，不断增强群众的获得感、幸福感、安全感，让全市人民在小康路上一户不落，日子一年比一年好。

　　二是坚持把提升发展的质量效益放到经济建设的首位。扎实推进质量变革、效率变革、动力变革，坚持“增量崛起”与“存量变革”并举、有效投资和消费升级共进、外向型经济和内生性发展同步，构建实体经济、科技创新、现代金融、人力资源协同发展的产业体系，不断提高资源节约集约利用水平和全要素生产率，引导经济朝着更高质量、更有效率、更加公平、更可持续的方向发展，加快实现常州制造向常州智造转变。

　　三是坚持把改革创新作为推动经济社会发展的根本动力。坚定不移向改革聚力、以创新开路、用开放引领，着力在产城融合综合改革、行政审批制度改革、投融资体制改革、开发区改革创新等领域取得新突破，在放宽市场准入、加快企业“走出去”步伐、促进投资贸易便利化等环节上取得新进展，在产业技术创新、重大平台建设、龙城英才引育、政府管理和服务创新等方面取得新业绩，努力使常州成为全省领先的创新创业高地。

　　四是坚持把提升城市竞争力、影响力和美誉度作为不懈追求。围绕建设具有突出竞争力和影响力的区域中心城市，在国家和省重大战略中谋求更大发展，持续优化城乡布局、不断完善综合交通、大力提升城市品质，扎实推进文化名城、旅游名城、运动之城、智慧之城建设，做美大运河文化带，打造美丽中国、健康中国、数字中国常州样板，提升全域文明城市建设水平，把常州建设成为“江苏中轴崛起”主枢纽和扬子江城市群重要战略支点。

　　五是坚持把工作推进的系统化、项目化、精细化作为重要方法。坚持谋在远处、干在实处，善于以系统化思维推动发展，注重以项目化措施抓好落实，自觉以精细化理念开展工作。认真研究制定重点工作推进的任务书、时间表、路线图，统筹推进重大项目建设与中小企业发展、工业经济“三位一体”转型与现代服务业创新升级、生态绿城提升与全域美丽乡村建设，持续办好“三优三安两提升”民生实事，推动常州发展行稳致远。

　　今年经济社会发展的主要预期目标是：地区生产总值增长7.5%;一般公共预算收入增长7.5%左右；规模以上工业增加值增长7.5%左右；固定资产投资增长7.5%左右；实际到账外资和进出口总额保持适度增长、质量有所提升；居民人均可支配收入增长与经济增长同步；研发经费支出占地区生产总值比重达到2.8%；全面完成省下达的节能减排任务。

　　为实现上述目标，我们将重点抓好八个方面工作：

　　（一）突出提质增效，加快产业转型升级

　　围绕建设现代化经济体系，坚持质量第一、效益优先，把培育新动能与改造提升传统动能紧密结合起来，推动产业迈上中高端，加快建设全国一流的智能制造名城。

　　1.持续聚焦重大项目建设。扎实开展“重大项目增效年”活动，突出项目投资强度、亩均产出效益等关键指标考核，引导重大项目建设向量质并举、效益引领转变。紧盯十大产业链和现代服务业，推进引资引技引智联动，大力招引一批旗舰型、税源型、富民型重大项目和优质中小项目。突出产业链招商、产业集群招商，运用产业投资基金、股权投资等市场化手段，加速产业和资本集聚。组织实施162个市重点项目，建设瑞声科技小镇、星宇车灯研发生产、华润分布式能源等一批重大项目。建立健全重大项目咨询论证和绩效评估机制，促进项目管理科学化、全过程。

　　2.大力推进中小企业发展。不断优化中小企业发展环境，推进落实各项扶持政策，扎实开展企业减负行动，着力降低制度性交易成本。全面实施企业金融顾问制度，积极开展政银企对接，推进金融产品和服务创新，强化金融对实体经济的精准支持。建立完善中小企业技术创新、质量品牌、征信等公共服务平台，更好满足企业发展需求。引导和支持中小企业“专精特新”发展，打造更多“隐形冠军”和行业小巨人。深入实施加快民营经济发展三年行动计划，加快“四转”步伐，推动民营经济做大做强。

　　3.深化工业经济“三位一体”。推进“中国制造2025”苏南城市群试点示范建设，争创“中国制造2025”国家级示范区。加强“云大物移智”与制造业深度融合，加快实施“千企上云”，推动制造业向高端化、智能化、服务化转变。扎实推进新一轮十大产业链建设，重点打造新能源汽车及汽车核心零部件、智能制造装备、以石墨烯为代表的碳材料等特色产业集群，十大产业链产值占规模以上工业比重达37%以上。加快传统优势产业改造升级，推进一批国家工业强基工程项目，建设一批智能车间、智能工厂，重点打造北自所常州基地等智能制造产业集聚区。全面落实企业创新“4个90%”要求，组织创新型企业实施100个左右重大科技项目，新增市级以上企业研发机构100家，不断壮大创新型企业集群。扎实开展质量提升行动，培育一批享誉全国的常州品牌，建设全国质量强市示范城市。

　　4.促进现代服务业创新发展。深入实施集聚载体建设、互联网平台经济发展等“五大工程”，着力发展科技服务、软件和信息技术等生产性服务业。大力发展现代金融，加快建设常州金融商务区，积极引育各类金融主体，主攻产业基金、股权交易等与产业深度融合的新金融，全力争取金融改革创新试验区。加快常州检验检测认证产业园、海峡两岸（常州）健康产业园等集聚区建设。以信息化、电商化、国际化、品牌化为方向推动专业市场转型。推进全域旅游发展，加快建设东方盐湖城茅山源境、孟河小黄山旅游度假区等旅游重点项目。

　　（二）坚持创新驱动，加快转换发展动能

　　坚定不移实施创新驱动战略，强化创新第一动力地位和作用，突出以科技创新引领全面创新，全力打造长三角特色鲜明的产业技术创新中心。

　　1.加速引育集聚人才。坚持实施人才强市战略，瞄准产业需求精准引进海内外精英人才、领军型创新创业人才和紧缺型实用型人才，全年引进领军型创新创业人才200名以上。加快完善人才保障体系，创新人才培养、引进、评价、流动和激励机制，推进产学研深度融合，更好发挥用人单位主体作用，打造最优人才生态圈。坚持以高端平台吸引高层次人才，建设南京航空航天大学天目湖校区，支持在常高校建设一流学科。激发和保护企业家精神，深入推进民营企业家队伍建设“百千万工程”，促进新生代企业家加快成长；实施“技能龙城”建设计划，大力弘扬劳模精神和工匠精神，培育更多精准匹配产业发展的“龙城工匠”。

　　2.加快创新载体建设。持续推进苏南国家自主创新示范区建设，大力引进国内外知名的研发机构，推动骨干企业创建国家重点实验室、工程实验室，支持政校企合作建立协同创新平台和创新联盟，加快在智能制造装备、石墨烯、光伏新能源、轨道交通等领域打造国家级、省级产业创新中心。充分发挥科教城“创新之核”辐射引领作用，大力推进内涵深化和创新发展，积极推进与中科院新一轮合作，加快江苏中科院智能院建设，高水平规划建设“科创走廊”。深入实施“创业常州”行动，重点在人工智能、大数据等领域打造一批特色众创空间。大力集聚创新资源，积极承接上海、深圳等地创新溢出效应，推进研发产业化，加快形成创新成果产业化高地。推进知识产权密集型企业和示范园区建设，万人发明专利拥有量超30件。

　　3.加快企业股改上市步伐。更大力度、更实举措推进企业股改上市，组建上市后备企业股权投资基金和上市公司产业并购基金，打造资本市场有影响力的“常州板块”。加大上市后备梯队建设力度，着力推进企业股改，全年新增股份制企业100家，新增上市企业10家以上。引导上市企业再融资，募集更多资金投向我市战略性新兴产业。支持上市企业以产业链为核心开展并购重组，利用资本市场实现裂变发展。

　　4.积极培育发展“四新经济”。以新技术、新产业、新业态、新模式引领产业升级，培育发展数字经济、分享经济、绿色经济、生物经济、创意经济等新业态，积极发展人工智能、大数据、印刷电子、传感器等新产业。开展“互联网+”“智能+”等产业融合行动，加快建设国家健康医疗大数据常州中心、航天云网工业大数据平台、中兴能源华东云计算基地等，培育大数据产业集群。积极构建常州特色军民融合深度发展格局，重点在航空航天、新材料、信息通信等领域培育军民融合特色产业集群。

　　（三）深化改革开放，拓展更多发展空间

　　把改革开放作为发展的根本之策，推进深层次的制度性变革，实施引领性的开放新举措，不断激发经济社会发展的动力和活力。

　　1.加快重点领域改革步伐。继续深化供给侧结构性改革，扎实推进“三去一降一补”，持续优化供给体系的质量和效率。深入推进产城融合综合改革，推动常金一体化发展，加快武进国家产城融合示范区和市级产城融合示范区建设步伐，尽快形成可复制可推广的制度成果。推进资源要素市场化配置改革，坚持以亩产论英雄、以质效配资源，实施工业企业资源利用综合评价，通过差别化价格、用地、排污和金融政策，倒逼落后产能退出和企业转型。加快投融资体制改革，创新财政投入方式，发挥财政资金“四两拨千斤”作用，加强社会事业等新领域债券发行和债转股工作，引导和支持社会资本参与公益性事业投资运营。

　　2.深化开发区改革创新。推动各开发区强功能、建生态、创优势，集中力量做强主导产业，培育特色创新集群，着力提升土地产出率、资源循环利用率、智能制造普及率，加快向现代产业园区转型。构建园区循环经济产业链，国家高新区和60%的省级开发区完成循环化改造。推动常州、武进国家高新区争先进位，推进常州、金坛、溧阳开发区升格为国家级，进一步加快天宁开发区东扩、钟楼开发区西进步伐。优化特色园区布局，推动资源整合和功能提升，把园区的承载力和竞争力做得更强。

　　3.提升经济国际化水平。把对外合作园区作为推动新一轮开放开发的重要引擎，推进中以、中德、中瑞等国际合作园区加快建设发展，推动苏澳合作园区建设取得新进展。促进外资质效提升，提高制造业和生产性服务业利用外资占比。加快复制自贸区改革试点经验，营造符合国际惯例的商务环境。大力发展跨境电商、进口交易中心等外贸新业态，积极扩大高新技术产品出口比重。支持企业深度参与“一带一路”建设，引导轨道交通、光伏、工程机械、纺织服装等优势产能更好地“走出去”。

　　（四）促进乡村振兴，推动城乡协调发展

　　坚持农业农村优先发展，全面实施乡村振兴战略，让农业成为有奔头的产业，让农民成为有吸引力的职业，让农村成为安居乐业的美丽家园。

　　1.加快发展现代农业。深化农业供给侧结构性改革，突出农业提质导向，提升发展特色林果、花卉苗木、种源农业等优势产业，实施十大现代农业产业园区和十大本土农产品品牌培育计划，深入开展国家农产品质量安全市创建。加快培育新型职业农民，发展壮大专业大户、家庭农场、农民专业合作社、农业龙头企业等新型农业经营主体，积极推动农业企业股改上市。促进一二三产业融合发展，推进新北区省级试点区建设，实施创意休闲农业提升行动，大力提升乡村旅游品质，推动农产品精深加工，着力打造优质农产品电商平台和农业电子商务发展集聚区。

　　2.大力推进城乡融合发展。建立健全城乡融合发展体制机制和政策体系，推动城乡产业发展、要素配置、基础设施、公共服务等全面一体化。深入实施省级经济发达镇和市级中心镇扩权强镇改革，建设一批“产镇人文”有机结合的新型小城镇。把特色小镇作为城乡融合发展的重要载体，加快打造一批省级以上特色小镇。统筹推进特色田园乡村、全域美丽乡村建设，重点建设“两区十点”示范、试点项目，传承提升农耕文明和乡村特色文化。加强乡村基础设施建设，着力建设“四好农村路”，推进农村生活污水治理工程，确保村庄污水设施覆盖率达70%以上。

　　3.持续深化农业农村改革。加快完善农村土地“三权分置”办法，引导发展多种形式的农业适度规模经营。加强农村产权流转交易市场建设，80%的行政村完成集体产权制度改革。在武进全域推行农村土地制度改革三项试点，争取到年底盘活集体建设用地2万亩、集体经营性建设用地出让金突破20亿元。持续推进“三资三化”专项提升行动，规范壮大村集体经济。

　　（五）提升功能品质，增强中心城市能级

　　以打造“江苏中轴崛起”主枢纽和扬子江城市群战略支点城市为目标，深入实施精美常州提升行动，推进城市更新，塑造城市特色，完善城市功能，着力提升城市集聚力、带动力和影响力。

　　1.加快基础设施建设。实施“东融西进、南接北联”区域大通道建设，开工建设溧广、苏锡常南部通道等高速公路和沿江城际铁路等重点工程，积极推进常泰过江通道、常溧高速北延、镇宣铁路等前期工作，建设天目湖通用机场，完成常州火车站南北站房一体化提升改造。加快常州综合港务区建设步伐，着力构建“三港一区”联动发展新格局。实施机场路快速化改造、340省道改扩建等工程，加快地铁1、2号线建设。积极推进与周边城市城际基础设施互联互通。加强市政公用设施建设，实施魏村水厂自来水深度处理工艺改造、天然气利用三期、江边污水处理厂四期等项目。加快推进新沟河、新孟河等水利工程建设。

　　2.彰显江南水乡风貌。高质量完成新一轮城市总体规划修编。坚持精明增长、精致建设，分类分区推进城市更新试点，实施中心城区72个低效用地改造提升项目，加快改造易淹易涝片区，有序推进老小区、城中村、背街小巷整治。加快城市新片区建设，加强城市设计，打造常州文化广场等一批“城市客厅”。加强历史文化名城保护，积极推进大运河文化带建设，打造高颜值的生态长廊、高品位的文化长廊、高效益的经济长廊。青果巷历史文化街区一批名人馆、张太雷纪念馆新馆等对外开放。

　　3.加强城市精细化管理。全面提升城市管理水平，深入开展城市环境综合整治，实施“精品街道”行动和市容专项整治工程，完善城乡一体长效管理办法，加强住宅小区物业管理。深化道路交通秩序综合治理，突出抓好工程运输车、电动自行车整治，加快城市公共停车场建设，促进社会停车资源共享，让市民出行更方便、更舒心、更安全。加快建设智慧城市，全面整合公共信息资源，加强大数据运用，打造智慧常州“一朵云”。

　　（六）优化生态环境，建设美丽宜居家园

　　牢固树立“绿水青山就是金山银山”的发展理念，把优良生态作为加速转型的“绿色引擎”，推进生产、生活、生态融合发展，为群众创造更加美好的宜居环境。

　　1. 打好污染防治攻坚战。扎实开展“263”专项行动，实施碧水蓝天净土工程。加强水环境治理，深入推进武进生态保护引领区建设、新北沿江生态保护工程，实施主城区“畅流活水”工程，严格落实“河长制”，确保29个国、省考核断面水质达标。强化大气污染防治，主要污染物排放总量削减完成省下达的任务，让市民拥有更多的蓝天。积极实施土壤污染防治行动计划，有序推进农用地及工业用地污染地块风险管控与治理修复。

　　2.打造生态绿城升级版。持续推进生态绿城“六大工程”，实施森林城市建设三年行动计划，启动国家生态园林城市创建工作，推进新老运河等重要河道两侧生态绿道、亲水廊道建设，实现增核6000亩，扩绿2000亩、联网50公里、成片造林1万亩，为市民提供更多优质的生态产品。将生态绿城建设与运动健康相结合，建设一批便民亲民的社区体育公园和健身步道，打造公园绿地“10分钟服务圈”，让公园动起来，让群众乐起来。

　　3.推动绿色发展常态化。全面开展国家生态文明示范市创建，支持溧阳、金坛参与宁杭生态经济带建设。持续推进节能减排，大力发展循环经济，推进武进、新北创建国家绿色生态示范城区，发挥武进绿色建筑产业集聚示范区效应，加快建设国家低碳试点城市。大力推行生活垃圾分类，积极探索垃圾处理长效机制，实现生活垃圾100%无害化处理。倡导和推进绿色出行，积极发展城市公共自行车网络，年内投放2万辆有桩单车。

　　（七）聚焦富民惠民，持续增加民生福祉

　　把富民作为一切工作的出发点和落脚点，不断满足人民日益增长的美好生活需要，持续增强群众的获得感、幸福感。

　　1.促进城乡居民增收。以创业带动更高质量就业，推进省级创业型街道（镇）、社区（村）、园区创建，保障好重点群体就业，建设就业创业先行区。大力发展富民产业，推动农业与旅游、文化、康养等产业深度融合，着力拓宽农民劳动收入和财产性收入渠道。坚持阳光扶贫、精准脱贫，实施新一轮茅山老区帮扶工程，确保全市70%以上农村建档立卡低收入农户人均年收入达到脱贫标准，经济薄弱村村级总收入有较快增长。创新扶贫协作模式，促进对口支援地区群众脱贫致富。

　　2.优化提升公共服务。以推进省定基本公共服务标准化建设为抓手，促进基本公共服务优质均衡。进一步优化教育规划布局，完成20个学校建设项目，深入推进教育集团化办学和义务教育学校标准化建设，不断扩大幼教资源有效供给。支持科教城建设省高水平高职教育园区、深化中德职业教育合作，积极创建国家产教融合试点城市。推进“健康常州”建设，深化公立医院改革，完善分级诊疗制度，做实做优医联体建设和家庭医生签约服务，支持中医药发展，市妇幼保健院、市一院钟楼院区等重点项目竣工投用。推进幸福康养示范工程，养老机构医疗服务基本实现全覆盖，标准化居家养老服务中心（站）城乡覆盖率分别达65%、38%。推进运动健康城市建设，新建一批百姓身边的体育场地设施，办好常州市第十五届运动会。

　　3.健全完善社会保障。继续提高企业退休人员基本养老金、城乡居民基础养老金等待遇标准，推进异地就医联网结算。扩大住房保障覆盖面，城镇中低收入住房困难家庭应保尽保，实现住房公积金扩面9万人，发展住房租赁市场特别是长期租赁，新开工各类棚户区改造2.2万套（户）、基本建成1万套（户）。实施精准救助保障工程，加强医疗救助与基本医保、大病保险制度的配套衔接，提高城乡低保、低保边缘家庭一次性生活补贴标准。

　　4.持续办好民生实事。围绕“七个有”要求，聚焦人民群众最关心最直接最现实的利益问题，坚持统筹谋划、量力而行，办实办好以“三优三安两提升”为重点的30件民生实事，让改革发展成果更多更公平惠及全体市民。突出抓好“一场”（停车场）“两道”（清洁河道、亲水步道）“三园”（社区体育公园、小游园、幼儿园）“四治”（易淹易涝片区治理、老小区安全隐患治理、农村生活污水治理、生活垃圾分类治理）工程，补齐民生短板，打造民生亮点。实施促进学生身心健康“六项行动”，努力让常州的孩子吃得好、长得壮、眼睛亮，健康快乐地成长。

　　（八）创新社会治理，共建共享美好常州

　　进一步加强和创新社会治理，在共建共治共享中保民安、促和谐，提高社会治理社会化、法治化、智能化、专业化水平。

　　1.夯实基层基础工作。加强社区治理体系建设，推行社区网格化治理模式，发挥“全要素网格”的治理效能，实现基层社会治理精细化。加快建设公共服务“一站式”受理平台和社会组织综合服务平台，改造提升30个社区综合服务中心。扎实推进全国和谐社区示范城市创建，支持新北区建设全国农村社区治理实验区，城市、农村和谐社区达标率分别达96.5%、86%。

　　2.提高社会文明程度。开展“爱常州”引领行动，创新提升道德讲堂，培育更多志愿服务品牌。实施文明城市建设三年提升行动，深化常态长效管理机制，力争全域文明城市建设水平全国领先。持续推进法治常州建设，扎实开展“七五”普法。大力推动文化事业和文化产业发展，加强公共文化服务体系建设，建成200个基层综合性文化服务中心，提升“文化100”等惠民工程，加快建设“书香常州”，打造“常州三杰”等红色文化品牌，推出一批文艺精品力作，为广大市民提供更丰富的精神食粮。

　　3.全力保障安全稳定。深化平安常州建设，完善现代治安防控体系，加强源头治理，推进联动共治，加强合成维稳中心、公共安全监管中心建设，提高应急管理能力，切实增强公众安全感。严格落实安全生产责任制，坚决防范和遏制重特大事故发生。加强食品药品安全监管，强化粮食安全责任。促进房地产市场平稳健康发展，做好金融等领域风险防控，确保社会和谐稳定。

　　与此同时，支持工会、共青团、妇联等人民团体工作，提高国防动员和双拥共建水平，加强外事侨台、档案方志、民族宗教、气象、老龄、残疾人事业和关心下一代等工作。

　　三、加强政府自身建设，建设人民满意政府

　　新的时代、新的征程，对政府工作提出了更高更严的要求。我们将以习近平新时代中国特色社会主义思想为指引，切实增强“四个意识”“八个方面本领”，努力建设人民满意的法治政府、服务型政府、创新型政府、廉洁政府。

　　（一）坚持依法行政。扎实推进政府立法，全面推行政府公职人员学纪学法制度，发挥好法律顾问作用，切实增强依法行政能力。深化综合行政执法体制改革，在辖市（区）层面整合组建5-7支综合执法队伍，常州经开区实现“一支队伍管执法”。强化行政权力监督，自觉接受市人大法律监督、工作监督和市政协民主监督，健全司法监督、社会监督和舆论监督机制。持续深化政务公开，重点推进重大建设项目批准和实施领域政府信息公开，切实保障人民群众的知情权、参与权和监督权。

　　（二）坚持科学决策。完善公众参与、专家论证和政府决定相结合的行政决策机制，以科学决策引领科学发展。推进新型智库建设，充分发挥发展战略特聘顾问的作用，支持民主党派参政议政，加强对事关常州长远发展的重大课题研究。坚持问政于民、问计于民、问效于民，畅通民意表达渠道，更好地听民声、聚民智。严格遵循重大行政决策法定程序，将决策责任追究制度与容错纠错机制相结合，既规范决策行为，又支持创新实践。

　　（三）优化政府服务。持续推进“放管服”改革，全力打造审批事项最少、办事效率最高、创新创业活力最强城市。市、辖市（区）组建行政审批局，推进武进区行政管理体制综合改革、新北区简政放权集成改革。用好政务服务“一张网”，积极推进“不见面审批”，继续优化和全面实施建设项目“五联合一简化”联审联办机制，推行企业投资项目信用承诺制，高质量实现“3550”目标。提升12345政府公共服务平台功能，市政务服务中心全面建立“一窗通办”新模式。严格落实首问负责制，加强重点工作督查，强化部门责任担当。完善政府目标管理体系，对所有财政专项资金项目实行绩效考核。牢固树立“服务就是义务、服务就是环境、服务就是效益”理念，用心当好服务企业、服务项目、服务群众的“店小二”，着力构建“亲”“清”新型政商关系，让最优服务成为常州发展永不褪色的金字招牌。

　　（四）加强廉政建设。坚定不移推进政府系统党风廉政建设，落实全面从严治党主体责任。健全廉政风险防控机制，强化对政府投资管理、公共资源交易等重点领域的审计和管控督查。管好用好公共财政资金，严控“三公”经费，一般性公共预算支出和政府专项资金压降10%。盘活用足国有资产，对市级党政机关及其下属单位用房实施集中统一管理。持之以恒正风肃纪，深入贯彻习近平总书记关于纠正“四风”不能止步、作风建设永远在路上的重要指示，认真落实中央八项规定实施细则，锲而不舍推动作风建设常态化、长效化，坚决防止不良风气反弹回潮，坚决查处隐形变异“四风”问题，树立勤政廉政的良好形象。

　　各位代表，新思想引领新实践，新时代开启新征程。让我们更加紧密地团结在以习近平同志为核心的党中央周围，以习近平新时代中国特色社会主义思想为指引，在省委、省政府和市委的坚强领导下，凝心聚力、开拓创新、真抓实干，为种好人民满意幸福树、建设“强富美高”新常州而不懈奋斗！
